# Botox in below knee amputation for the management of post-operative contracture: a systematic review

**DOI:** 10.11604/pamj.2024.47.26.42249

**Published:** 2024-01-23

**Authors:** Ahmmad Alfatih, Basil Ibrahim, Abduelraheim Abu, Maysa Hamza, Iman Hassan

**Affiliations:** 1 Galway Clinic, Galway, Galway, Ireland; 2 Manchester Foundation Trust, Manchester, United Kingdom; 3 Barts Health NHS Foundation, London, United Kingdom; 4 North Bristol Trust, London, United Kingdom; 5 Royal Liverpool Trust, Liverpool, United Kingdom

**Keywords:** Botox, botulinum toxin, below-knee amputation, post-operative contracture, muscle spasticity

## Abstract

During the 1970s, scientists first used botulinum toxin to treat strabismus. While testing on monkeys, they noticed that the toxin could also reduce wrinkles in the glabella area. This led to its widespread use in both medical and cosmetic fields. The objective of the study was to evaluate the potential use of Botox in managing post-operative contracture after below-knee amputation. We conducted a systematic review In Pubmed, Cochrane Library, Embase, and Google Scholar using the MESH terms Botox, botulinum toxin, post-operative contracture, amputation, and below knee amputation. Our goal was to evaluate the potential use of Botox to manage post-operative contracture in patients who have undergone below-knee amputation. Our findings show evidence in the literature that Botox can effectively manage stump hyperhidrosis, phantom pain, and jumping stump, but no clinical trial has been found that discusses the use of Botox for post-operative contracture. Botox has been used in different ways to manage spasticity. Further studies and clinical trials are needed to support the use of Botox to manage this complication.

## Introduction

### Uses of botulinum toxin in medicine

Botulinum toxin is a strong neurotoxin that causes paralysis by preventing the release of acetylcholine, a neurotransmitter, at the neuromuscular junction. This results in temporary chemical denervation of the muscle fiber that leads to partial paralysis and atrophy. It's worth noting that the effects of botulinum toxin are not permanent because the muscle gradually becomes reinnervated by nerve sprouting once the chemical denervation wears off [1].

Botulinum toxin has been safely and effectively used in medicine since its discovery to treat various medical conditions, including chronic migraine: it can be injected into the head and neck areas every 3 months to reduce the severity and frequency of migraine attacks and alleviate symptoms of chronic migraine [2]. Achalasia and other spastic muscle disorders of the oesophagus: Botulinum toxin injection is commonly used to treat achalasia and reduce oesophageal hiatus by decreasing lower oesophageal pressure [3].

Botulinum toxin is also considered as a potential solution for individuals experiencing overactive bladder and urinary incontinence when conventional medications prove ineffective. Furthermore, it is useful in treating erectile dysfunction by relaxing penile-resistant vessels and cavernosal muscle tone. Moreover, recent clinical trials have shown that it can also help alleviate sexual pain resulting from muscle spasms in pelvic floor muscle dysfunction, with an impressive 71% success rate [4]. Addition use of Botulinium toxin in Cerebral palsy-induced spasticity: botulinum toxin injection is used to enhance joint performance by decreasing spasticity-associated hypertonia and preventing lasting contracture and structural disintegration [5]. Treatment of anal fissure: topical botulinum toxin injection can improve symptoms associated with chronic anal fissure, with healing up to (74.9%) after one month [6]. Abdominal wall reconstruction: botulinum toxin injection into the transverse abdominis muscle can increase muscle length by about 3 cm and affect muscle thickness and hernia width [7].

## Methods

**Search strategy:** our literature review used a comprehensive search strategy to identify relevant studies in electronic databases. The primary databases utilized were PubMed, Cochrane Library, Embase, and Google Scholar. Medical Subject Headings (MESH) terms, including “Botox,” “botulinum toxin,” “post-operative contracture,” “amputation,” and “below knee amputation,” were used to capture a broad range of literature related to the use of botulinum toxin in managing post-operative complications after below-knee amputation.

**Inclusion and exclusion criteria:** articles included in the review met specific criteria to ensure relevance and quality. Inclusion criteria comprised articles focusing on the use of Botox or Botulinum Toxin in the context of below-knee amputation, postoperative complications, and related outcomes. Exclusion criteria involved studies not directly addressing the research question or lacking sufficient methodological rigor. Only studies available in English were considered.

**Database selection and data extraction:** the initial search revealed varying results across databases. Cochrane Library, Pubmed, and Embase yielded no results related to the research question. However, Google Scholar initially provided 311 results, of which 51 were assessed for relevance, and none were found to be directly applicable to the study. After refining the search terms to “spasticity” and “Botox,” Google Scholar produced 7380 results. Following relevance filtering, no relevant studies were identified. Cochrane Library initially showed 38 results, and when filtered for the last five years, seven studies were found. Of these, one study was relevant. Pubmed initially produced 1220 results, which were further refined to 395 for the last five years. After adjusting for publication type, 98 studies were identified. Upon closer examination, 13 studies were found to be relevant, and after removing duplicates, four papers were selected. Unfortunately, Embase provided only one study, which did not align with the focus of our review.

**Data synthesis and analysis:** data synthesis involves summarizing key findings from the selected studies. Due to the potential heterogeneity of study designs and outcomes, a narrative synthesis approach was employed. Themes and patterns emerging from the literature were identified, and findings were organized to address the research objectives.

## Results

We were unable to find any evidence in the literature to suggest that Botox has been used to treat muscle contracture following a below-knee amputation. However, there is evidence in the literature that suggests that Botox has been used to treat other conditions related to amputation such as stump hyperhidrosis, jumping stump, and phantom pain. It is noteworthy that, Botox has been used to treat spasticity post-stroke and cerebral palsy, which has a similar pathophysiology to muscular contracture post-operatively.

## Discussion

**Anatomical overview:** the thigh muscles are divided into three compartments: anterior, medial, and posterior. The femoral nerve innervates the anterior compartment, which includes the pectineus, sartorius, quadriceps femoris, and iliopsoas muscles. The quadriceps femoris is the primary knee extensor, while the sartorius muscle flexes the knee and hip joints, and the pectineus muscle flexes, adducts, and medially rotates the hip. The femoral artery enters the thigh to provide blood to the anterior compartment, and the deep femoral artery splits into two smaller branches that supply blood to nearby muscles. The external iliac lymphatic plexus drains the anterior thigh muscles and merges with the common iliac plexus before draining into the cisterna chyli and then the thoracic duct. The femoral nerve innervates the anterior compartment of the thigh, which includes the pectineus, sartorius, quadriceps femoris, and iliopsoas muscles. The femoral and deep femoral arteries provide blood supply to nearby muscles. The femoral nerve is responsible for motor and sensory functions in the anterior and medial legs. Occasionally, the pectineus muscle may also be innervated by the obturator nerve.

**Amputation:** an amputation is a medical procedure in which all or a portion of the limb is removed when saving the limb (upper or lower) is not feasible [8]. Leg amputees typically outnumber arm amputees which is more common within the younger age groups. Diabetes and age-related metabolic illnesses are two of the most frequent causes of limb loss due to peripheral vascular disease [9]. However, below the age of 60, trauma is most frequently the cause of limb amputation. Other causes include infection and malignancy [10]. The number of amputees who lose limbs in workplace accidents has significantly reduced over the past 20 years, whereas the number of amputees who lose limbs in accidents at home or while engaging in recreational activities has remained relatively stable [9]. Because the knee joint is preserved, Below-knee amputation (BKA) is linked to better rehabilitation outcomes than above-knee amputation (AKA). In contrast to Above-knee amputation (AKA), BKA typically has greater reoperation rates and worse overall healing rates. When BKA fails, it frequently calls for a further revision or perhaps an AKA [10].

**Below knee amputation indications:** below-knee amputations are done in critical situations where limb preservation is not possible, and when death is imminent. Definitive source control with debridement is essential. IV antibiotics play a crucial role in reducing morbidity caused by systemic bacterial infection. Urgent BKAs may be necessary if limb salvage fails. Full-thickness burns, severe or total neurovascular impairment, or permanent soft tissue abnormalities may require definitive BKA. Finally, non-septic patients with extensive non-healing tissue loss, multiple distal to mid-foot amputations with persistent infection, unreconstructedly vascular insufficiency with a non-healing ulcer, or lack of distal foot/ankle function with persistent pain may be candidates for elective BKAs [11].

**Below knee amputation contraindications:** vascular insufficiency at the anticipated amputation location is the primary reason to avoid non-urgent BKA. Preoperative checkup, ankle-brachial indices, Doppler, and oxygen pressure measurements in the toes should be done to assess the individual's condition. In cases of severe vascular insufficiency, bypass grafting or stent implantation may be necessary before performing a BKA [11]. Salvaging the knee joint in transtibial amputation is crucial for successful rehabilitation. This weight-bearing joint facilitates mobility and stability, allowing for functional activities. Preserving the knee joint offers mechanical advantages and enhances the ambulation potential of the patient. A study shows that healthcare professionals consider knee joint salvage to be vital for successful rehabilitation in transtibial amputees [12]. Patients undergoing a below-knee amputation are more likely to ambulate with a prosthesis than above-knee amputees [13].

**Advancement in lower limb amputation:** several techniques are available for lower-limb amputations, including transtibial for below-knee and transfemoral for above-knee amputations. The choice depends on factors such as the extent of injury or disease, overall health and fitness of the patient, and rehabilitation goals. Recent advancements include osseointegration, which involves implanting a metal rod into the residual limb to serve as a direct connection point for attaching the prosthetic limb. This technique has shown promising results in improving function and comfort for amputees [13] ambulation and better weight-bearing capabilities. Targeted muscle reinnervation is an advanced technique in amputation that involves reassigning nerves to nearby intact muscles, improving control and movement of prosthetic limbs and reducing complications. Rehabilitation and prosthetic considerations are crucial after lower limb amputation to help patients regain mobility and function [14]. A comprehensive rehabilitation program is key to a successful recovery. This usually involves a multidisciplinary team approach, including physiotherapists, occupational therapists, prosthetists, and psychologists.

**Prosthetic rehabilitation after amputation:** prosthetic rehabilitation is an essential component of treatment for individuals who have undergone lower limb amputation. The effectiveness of this rehabilitation is influenced by various factors, such as the amputation approach and residual limb characteristics. However, with continuous advancements in prosthetic technology, there is much hope for enhanced limb agility and functionality in the years ahead.

**Complications:** as is the case with all surgical procedures, complications can arise at any point during the amputation journey. These complications can be categorized as immediate or long-term and depend on factors such as the type of amputation, the overall health of the individual undergoing the procedure, and the surgical technique employed. Immediate complications may include wound infection, acute myocardial infarction, stroke, nosocomial infection, deep venous thrombosis, renal insufficiency, pneumonia, and even mortality [8,15]. Conversely, long-term complications can encompass phantom limb pain, stump pain, stump osteomyelitis, stump overgrowth, phantom limb sensations, painful bone spurs, hypertrophic scarring, and severe depression, however, physical, and psychological well-being has been shown to have a significant impact on quality of life [8].

**Contractures:** contracture is a common complication after below-knee amputation, characterized by the tightening of soft tissues. It can cause a limited range of motion and functional limitations. Lack of awareness regarding prevention methods and stump positions can contribute to its development [16]. Flexion contracture is a common complication after below-knee amputation. It can cause a limited range of motion and functional limitations. Lack of awareness regarding prevention methods and stump positions can contribute to its development. Trauma-related amputations may increase the risk of skin problems and flexion contracture at the knee joint due to the dominance of the hamstring muscles over the quadriceps [17]. Furthermore, above-knee amputations may also be at risk for contractures, specifically in the hip joint. The loss of adductor muscles and the dominance of hip abductors in above-knee amputations can lead to hip flexion, abduction, and external rotation contractures [18]. Above-knee amputations can lead to hip contractures due to the loss of adductor muscles and the dominance of hip abductors. To prevent contractures in below-knee amputations, various strategies like immediate use of a prosthesis, rigid dressings, and active range of motion exercises can be employed. However, the effectiveness of stretching in preventing or treating contractures is still unclear.

**Rehabilitation:** the selection of planned amputation sites is guided by several key factors, including considerations for patient survival, the alleviation of pain, improved prospects for prosthesis utilization, enhanced walking speed, and ultimately, a better quality of life, all of which contribute to more favourable rehabilitation outcomes [19]. Transitioning from inpatient rehab to home post-lower limb amputations is a demanding phase. The rehab process must equip patients with essential skills for daily tasks at home and in the community. Continuous community support is crucial for a seamless and successful transition [16]. Positive rehabilitation outcomes following amputation depend on factors such as pre-amputation ability, prompt admission, patient motivation, effective communication, and a multidisciplinary team [11]. Conversely, it is noteworthy that individuals with above-knee amputations often face challenges resulting in suboptimal rehabilitation outcomes. Specifically, those with above-knee amputations achieve the ability to ambulate outdoors with the aid of a prosthesis. In contrast, through-knee amputations have been reported in a wider spectrum of functional outcomes within this amputation category [20].

**Limitations:** this literature review has some limitations. The search was limited to articles published in English, which might introduce language bias. Additionally, the lack of clinical trials in the selected studies limited the ability to draw definitive conclusions about the use of Botox in managing post-operative contracture after below-knee amputation.

## Conclusion

Maintaining the range of motion after amputation surgery is crucial for successful rehabilitation and proper prosthesis fitting. Botox injections are effective in reducing muscle spasticity and preventing contractures, thus improving the range of motion in residual limbs. Combining Botox with conventional rehabilitation techniques can significantly enhance functionality and satisfaction in post-amputation patients. Botox has also shown good results as a prophylaxis for contracture. Evidence suggests that it can reduce upper limb spasticity and contractures after a stroke. The effects of Botox can last for approximately 12 weeks and do not interfere with the recovery of arm function. A clinical trial was conducted to study the treatment of spasticity in children with cerebral palsy using different doses of BTXA. The trial found that treatment with 8U/kg or 16U/kg abobotulinumtoxinA was more effective in reducing upper limb spasticity compared to the 2U/kg control dose. The therapeutic benefits of abobotulinumtoxinA, combined with HETP, were sustained with repeat treatment cycles. It is important to note that the effectiveness of Botox treatment can vary depending on individual factors such as the type of amputation, muscle involvement, and rehabilitation plan. Therefore, an individualized approach is necessary.

While Botox shows promise, there may be considerations regarding dosage, timing of injections, and potential side effects that need to be carefully managed. Botox works by temporarily obstructing nerve signals targeting specific muscles, leading to muscle relaxation. This mechanism is of significant value in the prevention of muscle overactivity, which can result in contractures. Literature suggests that Botox may not only be effective in treating contractures but also serve as a preventative measure. To gain a deeper understanding of the potential benefits and risks of utilizing Botox as a post-operative contracture treatment or prophylaxis following amputation, it is proposed to conduct clinical trials. Such an undertaking could lead to improved treatment options for individuals who have undergone amputation.

### What is known about this topic

- *Botox has been used in humans for decades, with efficiency, it has been used to treat muscle spasticity in patients post-stroke, and cerebral palsy;*
- *It has been used before in amputation patients for the management of conditions such as jumping stump, phantom pain, and stump hyperhidrosis*.

### What this study adds

- *This study discussed the possibility of using Botox in below knee amputation patients to manage post-operative contracture, additionally, it also, as mentioned in the article, has prophylactic properties*.
